# 2021 List of Reviewers

**DOI:** 10.1117/1.NPh.9.1.010102

**Published:** 2022-01-21

## Abstract

Thanks to reviewers who served Neurophotonics in 2021.

*Neurophotonics* would like to sincerely thank the following individuals who served as reviewers in 2021. The success of our publication hinges on the voluntary contributions of time and energy put forth by these professionals.

Androu Abdalmalak
Anna Letizia Allegra Mascaro
Juanita Anders
Kamran Avanaki
Wesley Baker
Adam Bauer
Bernhard Baumann
Addison Billing
Borja Blanco
Pablo Blinder
Stephanie Bonney
Oliver Bracko
Sabrina Brigadoi
Marco Capitanio
Jaepyeong Cha
Siyu Chen
Antonio Chiarelli
Nicole Comfort
Irene Costantini
Joao Couto
Alberto Dalla Mora
Hamid Dehghani
Michele Desjardins
Shinghua Ding
Patrick Doran
Konstantin Doubrovinski
Patrick Drew
Jessica Dubois
Anirban Dutta
Adam Eggebrecht
Christian Eggeling
Tae Joong Eom
Sinem Erdogan
Cornelius Faber
Sergio Fantini
Jessica Filosa
Rodrigo Forti
Clement Francois
Tsukasa Funane
Yuanyuan Gao
Jessica Gemignani
Adam Gibson
Natalie Gilmore
Albert Gonzales
Gorm Greisen
Soren Grubb
Edgar Guevara
Christian Henneberger
David Highton
Keum-Shik Hong
Song Hu
Jeffrey Iliff
Xavier Intes
Jana Kainerstorfer
Elisa Kallioniemi
Georgios Keliris
Caroline Kelsey
Tiffany Ko
Kijoon Lee
Adam Liebert
Chao Liu
Gangjun Liu
Chris McKnight
Shalin Mehta
Guanghan Meng
Miriam Menzel
Arcangelo Merla
Rickson Mesquita
Daniel Milej
Muge Molbay
Yukifumi Monden
Laleh Najafizadeh
Andrew Nelson
Thien Nguyen
Haijing Niu
Jack Noah
Sergio Novi Junior
Yumie Ono
Felipe Orihuela-Espina
Antonio Ortega-Martinez
Patricia Pais Roldan
Ashwin Parthasarathy
Gulcin Pekkurnaz
Marcela Pena
Stephane Perrey
Thao Pham
Luca Pollonini
Vidhya Rangaraju
Darren Roblyer
Sara Sanchez-Alonso
Hendrik Santosa
Angelo Sassaroli
Hiroki Sato
Joao Sato
Giovanni Scavariello
Christopher Schaffer
Felix Schlegel
Felix Scholkmann
Anna Schueth
Yu Shang
Noam Shemesh
Andy Shih
Shy Shoham
Yousheng Shu
Julie Siegenthaler
Spencer Smith
Janet Sorrells
Kurt Steinmetzger
Jillian Stobart
Jason Sutin
Kevin Takasaki
Jianbo Tang
Qinggong Tang
Tong Boon Tang
Alexander Thompson
Vladislav Y. Toronov
Cam Ha Tran
Julie Uchitel
Hana Uhlirova
Gregoire Vergotte
Ernesto Vidal Rosas
Karthik Vishwanath
Flavia Vitale
Alexander von Luehmann
Benquan Wang
Jack Waters
Ian Winship
Martin Wolf
Yujia Xue
Toru Yamada
Ji Yi
Qiang Yi
Zhen Yuan
Meryem Yucel
Xian Zhang

